# Breastfeeding initiation and duration and acute otitis media among children less than two years of age in Jordan: results from a case–control study

**DOI:** 10.1186/s12887-022-03427-7

**Published:** 2022-06-28

**Authors:** Furat K. AL-Nawaiseh, Madi T. Al-Jaghbir, Mohammad S. AL-Assaf, Hala K. AL-Nawaiseh, Majdi. M. Alzoubi

**Affiliations:** 1 Department of Family and Community Medicine, School of Medicine, The University of Jordan, Amman, Jordan; 2 Department of Ears, Nose and Throat, King Hussein Medical Centre (KHMC), Amman, Jordan; 3 Department of Nutrition and Food Technology, School of Agriculture, The University of Jordan, Amman, 11942 Jordan; 4 Department of Nursing, School of Nursing, Al Zaytoonah University of Jordan, Amman, Jordan

**Keywords:** AOM, Exclusive Breast feeding, Artificial feeding, Children, Jordan

## Abstract

**Background:**

Acute otitis media (AOM) is one of the most common infectious diseases that affects children. Breastfeeding has been linked to a lower risk of AOM in the first three years of childhood. The aim of this study was to identify the association between exclusive breastfeeding and the development of acute otitis media (AOM) and investigate the influence of breastfeeding duration on the presence of AOM.

**Methods:**

In a retrospective case–control study, a sample of 98 children (cases) who were diagnosed with AOM and 98 children (controls) who were not diagnosed with AOM and were younger than two years old were selected from the Jordan University Hospital. Medical records were used to identify children with AOM. For both the case and control groups, the children’s mothers completed a self-administered questionnaire about factors linked to the incidence of AOM.The type of feeding and the duration of breastfeeding were assessed using a validated questionnaire.

**Results:**

The data indicated that among children who developed AOM, 23.5%were artificiallyfed, while 22.4% and 13.3% were exclusively breastfed for 3 months and 6 months, respectively. Approximately 70.7% of the children without AOM were exclusively breastfed for 6 months, compared with only 29.3% of the children without AOM who were exclusively breastfed for 3 months.Logistic regression revealed that nonexclusive breastfeeding, exclusive breastfeeding for 3 months, and exclusive breastfeeding for 6 months were protective factors against AOM (OR = 0.23, 0.18, and 0.25, respectively; *P* < 0.05). A short duration of exclusive breastfeeding was considered a risk factor for the development of AOM (OR = 1.7, *P* < 0.05).

**Conclusions:**

The escalation of AOM is tightly connected to the early introduction of formula feeding in the first six months of life. Breastfeeding had a protective impact on the occurrence of AOM. Understanding factors that are associated with the occurrence of AOM in children may support the role of public health institutions and primary health care in the prevention and reduction of AOM episodes and the need for national health strategies to promote breastfeeding.

## Background

Acute otitis media (AOM) is one of the most common infectious diseases that affects children [1]. It affects over 70%of children before their second birthday, and it is considered one of the most common reasons for children to take antibiotics in developing countries, where AOM is estimated to affect more than 60% of children under the age of one year and more than 80% of children under the age of three years [1, 2]. However, exclusive breastfeeding (EBF) has protective effects for range of infectious diseases, including OM [1]. It is the most effective feeding method to reduce the risk of mortality and morbidity among children and to provide them with sufficient nutritional requirements to grow and develop in optimal conditions [3–5]. Moreover, breast milk contains host defence factors that inhibit the major cause of OM, which is Haemophilus influenza, and lower respiratory tract infections during childhood [6].

Based on the World Health Organization (WHO) definition,EBF has been defined as when infants receive only breast milk and accept the use of rehydration salts, drops, syrups, vitamins, minerals or medicines [5]. However, the short- and long-term benefits of breastfeeding for the protection of infants are globally acknowledged [5].Data from the Jordan Population and Family Health Survey (JPFHS) indicated that the percentage of children who are exclusively breastfed declines with age, from 43% among children under the age of 2 months to 11% among children aged 4–5 months [7]. The lack of sufficient studies related to the association between exclusive breastfeeding and the development of AOM among children younger than 2 years of age in Jordan requires further studies, which can provide grounds to draft and implement a health care plan that can promote exclusive breastfeeding to reduce the risk of AOM among young children and decrease its recurrence. In addition, it will decrease the economic burden as a result of the decreased use of antibiotics.

## Methods

### Design

A retrospective case–control study was conducted from November 2020 to January 2021 to examine the possible association between exclusive breastfeeding and AOM;a group of children diagnosed with AOM who were aged from zero-two years and a group of apparently healthy children who had no previous episodes of AOM in Jordan University Hospital (JUH), Amman, Jordan during the period from 1/1/2017 until 31/12/2020 were compared.

### Participants

The study protocol was approved by the authorized ethics committees at the University of Jordan and ethical approval was obtained from the Scientific Research and Ethical Committee of JUH. The population sample of the current study consisted of 98 cases (children with AOM) at JUH and 98 controls (children without AOM). Data were collected using a self-administered questionnaire and face-to-face interview (FTF) between the researcher and mothers of children visiting the General Pediatrics Clinic at JUH; this was performed for the first 10 cases and 10 controls after checking the diagnoses documented in their medical records. After this, the circumstances of the COVID-19 pandemic forced us to complete the data collection using direct telephone calls with the children’s mothers. Therefore, we collected the remaining 176 participants, with 88 cases and 88 controls.Children whose mothers wanted to take part in the study were screened to determine whether they met the predetermined inclusion and exclusion requirements. Mothers were provided with an information kit containing a brief explanation of the nature, objectives, and main method of the study. Before participating in the study, informed consent was obtained from a parent for study participation. Eligibility for the study was determined using the following criteria:

#### Inclusion criteria

1. Children who were diagnosed with AOM (cases) at JUHwhen they were less than two years old, during the period from 1/1/2017 until 31/12/2020.
2. Children who were apparently healthy (controls) and were not diagnosed with AOM at JUH when they were less than two years old, during the period from 1/1/2017 until 31/12/2020.

#### Exclusion criteria

At the time of recruitment, children with the following characteristics were deemed ineligible to participate in the study:

1. Children with missing AOM data.
2. Children born before the 34th week of pregnancy, due to an elevated risk of infection [8].
3. Children with lip-palate malformation, down syndrome, and any craniofacial deformity due to an increased risk of OM [8].

### Sample size

The target sample size was estimated using G. power 3.0.10 and an effect size of 0.3, with a power of 80% at a significance level of α = 0.05for statistical significance [9]. Based on this, the estimated total sample size was at least 143 children who were younger than 2 years and were visiting the General Pediatric Clinic at JUH; the children were classified into the case group or control group (72 children per group were required). After following the data collection procedure, 196 children participated in this study, with a response rate of 85%, which was considered a high response rate.

### Data collection procedure

The data were collected from the General Pediatric Clinic at JUH and medical records, and we used a self-administered questionnaire format for data collection. The children’s mothers were interviewed by the researcher through face-to-face and telephone interviews to collect data, including sociodemographic data, infant feeding data, and passive smoking data. Medical histories were obtained through the children’s medical records at JUH. Information regarding the infant and family histories and infant feeding patterns were obtained using a structured validated questionnaire that was completed by the children’s mothers.

### Study tool

The required data were collected using appropriate measurement tools that were obtained from the previous literature. The related questions were translated into Arabic by the researcher and then back translated by another person to ensure proper understanding of the meaning of each question. The questionnaire was pretested with40 participants who were excluded from the main study. We used the same exclusion and inclusion criteria for the actual study. Cronbach’s alpha was 0.70, showing evidence of the questionnaire’s reliability and internal consistency [10].

#### Demographic data

Information on participant characteristics was collected in the demographic data section of the self-report questionnaire to describe the sample. Were asked about their sex, their educational level, and their child’s age.

#### Feeding data

The questionnaire included questions about the type of feeding (exclusive breastfeeding, artificial feeding, breastfeeding and artificial feeding), duration of exclusive breastfeeding (artificial feeding, three months, more than six months), and barriers related to breastfeeding.

The definitions were explained for the participants as follows:

- Artificial feeding: Infants are fed only using a breast milk substitute [11].
- Breast milk substitute: Any food being marketed or otherwise represented as a partial or total replacement for breast milk, regardless of whether it is suitable for that purpose [12]
- Breastfeeding: The child has received breast milk directly from the breast or milk that was expressed [13]
- Exclusive breastfeeding: No other food or drink, not even water, except breast milk (including expressed milk or milk from a wet nurse) for the first 6 months of life, but the infant is allowed to receive ORS, drops and syrups (vitamins, minerals and medicines) [14]

#### AOM data

- The researcher visited the General Pediatric Clinic to review medical records from 1/1/2017 to 31/12/2020, and then we collected cases who were diagnosed with AOM when they were less than 2 years old. Additionally, we collected controls after reviewing their records, which showed no evidence of previous AOM episodes during the first 2 years of life. We conduct our study based on the definition of AOM [15] and we depend on reviewing the cases with documented bulging and redness of tympanic membrane along with acute onset of fever in addition to the presence otitis media with effusion using an objective test which is the tympanometry.
  In the analysis, the following conditions were used to diagnose OM (AOM and OME):
- AOM: eardrum inflammation with anabrupt onset of symptoms (fever, irritability, or earache) and signs (fluid level, bubbles, hypervascularity, withdrawn tympanic membrane) as documented by pneumatic otoscopy and/or tympanometry [15].
- Acute otitis media (AOM): Rapid onset of signs and symptoms of inflammation in the middle ear [16].
- Recurrent AOM: Three or more well-documented and separate AOM episodes in the preceding 6 months, or four or more episodes in the preceding 12 months with more than one episode in the past 6 months [16]

### Statistical analysis

The Statistical Package for the Social Sciences (SPSS version 23) was used to analyse the variable (breastfeeding). Descriptive analysis was carried out for sociodemographic data. Frequency tables were constructed as appropriate. The chi-square test (χ2), the independent t test, and logistic regression were used to test for statistical significance, and differences were considered significantly different at a *P value* < 0.05.

## Results

### Sociodemographic characteristics

The final sample consisted of 98 children (cases) who were diagnosed with AOM and 98 children (controls) who were not diagnosed with AOM at Jordan University Hospital (JUH) in Jordan. The sociodemographic characteristics, including sex, age of the child, and the education level of the mother, are presented in Table 1. The data were compared between the case group (children with AOM) and the control group using the chi square test and independent t test. According to the results of the Chi square test, it was found that there were no significant differences between the case and control groups for sex (*p* = 0.061). The frequency of males among the case group was 63.3%, while in the control group, it was 50%; however, there was no statistically significant difference. The results of the Chi square test for the mother’s education level indicated that the case and control groups differed significantly (χ2 = 15.199, *p* = 0.004). The frequencies of higher education categories, including bachelor’s (60.2%) and postgraduate (16.3%) degrees, were higher in the control group than in the case group (51% and 7.1%, respectively). However, the number of mothers with a high school education was higher among the case group (24.5%) compared to the control group (6.1%). The mean age (months) ± standard deviation (SD) was 21.26 ± 4.3 months among the cases compared to 19.35 ± 4.75 months among the control participants (Table 1).

**Table 1:** Sociodemographic Characteristics of the Study Population Stratified by Group

Variable	Level	Cases	Controls	*p*- value
*Sex	Female	36(36.7)	49(50)	0.061
	Male	62(63.3)	49(50)	
**Age (months)		21.26 ± 4.3	19.35 ± 4.75	0.04
*Level of Education	Preparatory	6(6.1)	5(5.1)	0.004
	High school	24(24.5)	6(6.1)	
	University college	11(11.2)	12(12.2)	
	Bachelor’s	50(51)	59(60.2)	
	Postgraduate	7(7.1)	16(16.3)	

^*^Data are presented as the frequency (percentage) and are considered statistically significant at *p* < 0.05

^**^ Data are presented as the mean ± SD and are considered statistically significant at *p* < 0.05

### Types of feeding among the study participants

Table 2 presents the types of feeding among the study participants. The data indicated that 23.5% of the children who developed AOM were artificially fed, while 40.8%, 22.4%, and 13.3% of the children who developed AOM were nonexclusively breastfed, exclusively breastfed for 3 months and exclusively breastfed for 6 months, respectively.

**Table 2:** Types of Feeding among the Study Participants

Variable	Level	Cases	Controls	*p-* value
**Type of feeding**	**Nonexclusive**	40 (40.8)	23 (23.5)	0.000
	**Artificially Fed**	23 (23.5)	17 (17.3)	
	**Exclusively breastfed-3 months**	22 (22.4)	17 (17.3)	
	**Exclusively breastfed -6 months**	13 (13.3)	41 (41.9)	

^*^Data are presented as the frequency (percentage) and are considered statistically significant at *p* < 0.05

### Association between the type of feedings and the development of acute otitis media (AOM)

The data indicated that 23.5%of the children who developed AOM were artificially fed, while 40.8%, 22.4%, and 13.3% of the children who developed AOM were nonexclusively breastfed, exclusively breastfed for 3 months and exclusively breastfed for 6 months, respectively. Pearson’s chi-square test revealed a significant association between the type of feedings and the presence of AOM (Χ2(3) = 20.647, *p* < 0.05; Table 3).The categorical logistic regression model revealed that nonexclusive breastfeeding, exclusive breastfeeding for 3 months, and exclusive breastfeeding for 6 months were protective factors against AOM (OR = 0.23, 0.18, and 0.25; respectively, *P* < 0.05; Table 3).

**Table 3:** AOM among Children Younger than 2 Years of Age in Jordan/JUH who were Artificially Fed, Nonexclusively Breastfed, Exclusively Breastfed for 3Months, and Exclusively Breastfed for 6Months

Type of Feeding	Cases	Controls	Chi-Square	*P value*	Odds Ratio (OR)
Artificially Fed	23 (23.5)	17 (17.3)	20.647	0.000	
Nonexclusively Breastfed	40 (40.8)	23 (23.5)			0.234
Exclusively Breastfed-3 months	22 (22.4)	17 (17.3)			0.182
Exclusively Breastfed -6 months	13 (13.3)	41 (41.9)			0.245
Total	98	98			

^*^Data are presented as the frequency (percent %)

The difference is significant at the 0.05 level

### Association between the duration of breastfeeding and the development of Acute Otitis Media (AOM)

The presence of AOM among participants who were exclusively breastfed for 3 months vs. 6 months is reported in Table 4.The data indicated that 41 (70.7%) of the children who did not develop AOM were exclusively breastfed for 6 months, compared with only 17 (29.3%) of the children who did not develop AOM who were exclusively breastfed for 3 months. Pearson’s chi-square test revealed a significant association between the duration of breastfeeding and the presence of AOM (Χ^2^(1) = 10.08, *p* < 0.05; Table 4). Logistic regression revealed that a short duration of exclusive breastfeeding was considered a risk factor for the development of AOM (OR = 1.7, *P* < 0.05, Table 4).

**Table 4:** AOM among Children Younger than 2 Years of Age in Jordan/JUH who were Exclusively Breastfed for 3Months and Children who Exclusively Breastfed for 6Months

Type of Feeding	Cases	Controls	Chi-Square	*P value*	Odds Ratio (OR)
Exclusively Breastfed-3 months	22 (62.9)	17 (29.3)	10.088	0.001	1.7
Exclusively Breastfed -6 months	13 (37.1)	41 (70.7)			
Total	35 (37.6%)	58 (62.4%)			

^*^Data are presented as the frequency (percent %)

The difference is significant at the 0.05 level

### Association between pumped breast milk and the development of acute otitis media (AOM)

The data indicated that 37.5% of the children who developed AOM received pumped breast milk compared to 90.8%of the children who did not develop AOM and did not receive pumped breast milk. Pearson’s chi-square test revealed a significant association between receiving pumped breast milk and the presence of AOM (Χ^2^(1) = 16.721, *p* < 0.05; Table 5). Logistic regression revealed that receiving pumped breast milk was considered a risk factor for the development of AOM (OR = 5.91, *P* < 0.05, Table 5).

**Table 5:** Effect of Receiving Pumped Breast Milk on the Presence of AOM among Children Younger than 2Years of Age in Jordan/JUH

Receiving pumped breast milk	Cases	Controls	Chi-Square	*p value*	Odds Ratio (OR)
No	45(62.5)	69(90.8)	16.721	0.00	5.91
Yes	27(37.5)	7(9.2)			
Total	72	76			

^*^Data are presented as the frequency (percent %)

The difference is significant at the 0.05 level

## Discussion

Breastfeeding has been linked to a lower risk of AOM in the first three years of life [17]. Otitis-prone children often have a defective or immature antibody response, especially a reduced level of immunoglobulin G2 (IgG2) [2]. Moreover, breast milk contains host defence factors that inhibit a major cause of OM (Haemophilus influenza) and lower respiratory tract infections during childhood [6].Findings from the current case–control study indicated that 23.5%of the children who developed AOM were artificially fed. However, 40.8%, 22.4%, and 13.3% of the children who developed AOM were among the nonexclusive breastfeeding, exclusive breastfeeding for 3 months, and exclusive breastfeeding for 6 months groups, respectively. Similarly, it was reported that breastfeeding through the first three months of life reduces the risk of developing AOM by 13% [18]. Our data agreed with the results of a study among 1963 participants that found the recurrence of OM (more than three episodes during the past 3 months) in children who were bottle-fed compared with children who were breastfed for more than six months (OR = 2.30) [19]. Our data were also consistent with data from a multicentre cohort study in which 2258 children aged 0 to 71 months from five Eastern European countries were enrolled [20]. However, a reduced adjusted OR of 0.19 for AOM was reported for breastfed children compared with children who were not breastfed [20]. Current findings showed a significant association between the type of feedings and the presence of AOM (*p* < 0.05). Our data indicated that nonexclusive breastfeeding, exclusive breastfeeding for 3 months and exclusive breastfeeding for 6 months were protective factors against AOM compared with formula feeding (*P* < 0.05). The data indicated that breastfeeding was a protective factor against infections, unlike formula feeding, which placed children under immunodeficiency status and therefore led to a greater risk for infections [21]. Current data showed that 70.7% of the children who did not develop AOM were exclusively breastfed for 6 months, while 29.3%of the children who did not develop AOM were exclusively breastfed for 3 months. Our results showed a significant association between the duration of breastfeeding and the presence of AOM (*p* < 0.05). The current findings indicated that a short duration of exclusive breastfeeding was considered a risk factor for the development of AOM (OR = 1.7, *P* < 0.05).

Breastfeeding protected infants in industrialized countries against AOM, and a dose/duration response effect was reported [2]. In addition, a clear connection between breastfeeding and a lower risk of AOM within the first two years of life was found [18]. It was estimated that the average reduction of AOM risk among all categories of breastfeeding was 30–40 [18]. Moreover, our findings agreed with the findings of the Scotland study, which reported a significant protective effect of breastfeeding among children who were six months of age and younger than six months of age [22]. Moreover, these children had an OR of 2.13 if they were formula fed compared with those who were exclusively breastfed [22]. However, no significant association was documented among children older than six months of age or with those receiving mixed feedings [22]. It was reported that the recurrence of OM was greater among children who were breastfed for 4–5 months compared with those who were breastfed for more than six months (OR = 1.95) [19]. The data indicated decreased respiratory tract infections, especially the recurrence of OM, in children who were fully breastfed for more than six months compared with those who were breastfed for only four months [19].Moreover, a lower odds of acquiring AOM was reported among children who breastfeed for 0–2 months of life, but not beyond [23]. It wasfound that breastfeeding reduced the probability of AOM, recurrent AOM, and AOM with effusion even if it was in short duration or nonexclusive breastfeeding compared to using formula [23, 24]. However, several studies reported a lower risk of AOM among children up to two years of age who were exclusively breastfed for at least 6 months [8]. Consistent with our findings, data from a cohort study based on the US IFPS II population (infant feeding practices study II) with a 6-year follow-up reported a statistically significant association between exclusive breastfeeding and a reduced risk of OM; however, the findings were statistically significant when compared with ≥ 6 months of exclusive breastfeeding with0–3 months of exclusive breastfeeding [25]. Our data were similar to another study that found that among 1963 participants, the recurrence of OM (more than three episodes during the past 3 months) was higher in children who were bottle-fed compared with children who breastfed for more than six months (OR = 2.30) [19]. Moreover, a reduced OR of 0.40 for AOM was found in children who exclusively breastfed for at least 3 months compared with children who exclusively breastfed for less than 3 months [26]. Several researchers documented a protective effect of breastfeeding against different OM-related outcomes [18, 22]. Breastfeeding for 6 months or more was a protective factor against AOM compared with breastfeeding for less than 6 months [2, 26, 27]. A limited number of studies have estimated the protective effects of breastfeeding for longer than 6 months against AOM [25]. Onlyone study showed that breastfeeding for more than 9 months was beneficial compared to breastfeeding for less than 3 months [25]. Breastfeeding was found to protect against OM for the first two years, with defence being better for those who were primarily breastfed and those who were breastfed for a long period of time (6 months) [18, 26, 28, 29].The initiation of bottle feeding during the first six months of life was linked to an elevated risk of OM compared to six months of sole breastfeeding [21]. Our findings indicated that 37.5% of the children who developed AOM received pumped breast milk compared to 90.8%of the children who did not develop AOM and did not receive pumped breast milk (*p* < 0.05). The current data showed that receiving pumped breast milk was a risk factor for the development of AOM (OR = 5.91).Our findings agreed with other data that indicated that there was a strong association between the occurrence of AOM and bottle feeding inthe first 6 months of 2 life (OR = 2.93, *p* value < 0.05) [30]. It was documented that early introduction of cow’s milk was a risk factor for developing OM [31]. Introducing cow’s milk before the age of 12 months is not recommended because it is associated with immunodeficiency [31].Furthermore, the microbial composition of cow's milk can encourage pathogen colonization, resulting in chronic otitis media with effusion (COME) [32]. There were no major variations in the number of episodes of AOM between infants who were exclusively breastfed and those who were mixed-fed in the first 12 months of life, according to a report [33]. A slightly increased risk was reported among children who were exclusively breastfed compared with those who received mixed feedings (OR = 1.28). However, there was no significant difference in the risk of frequent OM between children who were exclusively breastfed for 6–7 months compared with those who were exclusively breastfed for 3–4 months in the first 12 months of life [34]. In a study conducted to investigate the effect of the mode of milk delivery (feeding at the breast, expressed milk feeding, or formula feeding) on the risk of OM, a significantly reduced OR of 0.96 for diagnosed OM was found in children who were fed “at the breast” for 1 month compared with others [34].

## Conclusions

In conclusion, the current study showed that the escalation of AOM was connected to the early introduction of formula feeding in the first six months of life. Moreover, breastfeeding had a protective effect on the occurrence of AOM, which was affected by the duration of breastfeeding. Exclusive breastfeeding for the first 6 months of life was more protective than breastfeeding for the first 3 months of life. Therefore, public health decision-makers should promote and support breastfeeding through the first six months of life through awareness campaigns, supporting mothers and assessing the presence of AOM in children under the age of two years to implement therapeutic care. The current study has a few limitations. First, the study was conducted during the COVID-19 pandemic, which disserved the meetings with mothers, and more time was needed to complete the data collection. Second, the current study was based on a relatively small sample of children. Therefore, the generalization of the current findings outside of the current population is limited. Third, the current study was predisposed to recall bias, as it was a retrospective study. Finally, the percentage of females in the control group was equal to the percentage of males. However, it was not significant compared to the case group.

## Data Availability

The datasets used and/or analysed during the current study are available from the corresponding author upon reasonable request.

## Abbreviations

AOM: Acute otitis media
COME: Chronic otitis media effusion
EBF: Exclusive breastfeeding
FTF: Face-to-face interview
JPFHS: Jordan Population and Family Health Survey
JUH: Jordan University Hospital
OM: Otitis media
OME: Otitis media effusion
WHO: World Health Organization

## References

[1] Lodge CJ, Bowatte G, Matheson MC (2016). The role of breastfeeding in childhood otitis media. Curr Allergy Asthm.

[2] Salah M, Abdel-Aziz M, Al-Farok A (2013). Recurrent acute otitis media in infants: analysis of risk factors. Int J Pediatr Otorhinolaryngol.

[3] Laugen CM, Islam N, Janssen PA (2016). Social support and exclusive breast feeding among Canadian women. Paediatr Perinat Epidemiol.

[4] Victora CG, Bahl R, Barros AJ (2016). Breastfeeding in the 21st century: epidemiology, mechanisms, and lifelong effect. Lancet.

[5] Martines F, Salvago P, Ferrara S (2016). Factors influencing the development of otitis media among Sicilian children affected by upper respiratory tract infections. Braz J Otorhinolaryngol.

[6] Hokama TO, Sakamoto RY, Yara AS (1999). Incidence of Haemophilus influenzae in the throats of healthy infants with different feeding methods. Pediatr Int..

[7] Jordan Population and Family Health Survey 2017–2018. http://dosweb.dos.gov.jo/products/dhs2017-2018/. Accessed 12 Feb 2021.

[8] Kørvel-Hanquist A, Djurhuus BD, Homøe P (2017). The effect of breastfeeding on childhood otitis media. Curr Allergy Asthm.

[9] Erdfelder E, Faul F, Buchner A (1996). GPOWER: A general power analysis program. Behav Res Methods.

[10] Polit DF, Beck CT (2010). Generalization in quantitative and qualitative research: Myths and strategies. Int J Nurs Stud.

[11] Infant and young childfeeding. https://data.unicef.org/topic/nutrition/infant-and-young-child-feeding/. Accessed 15 Sep 2021.

[12] Marketing of Breast-milk Substitutes: National Implementation of the International Code — STATUS REPORT2020. https://www.unicef.org/media/69641/file/Marketing-of-breast-milk-substitutes-status-report-2020. Accessed 23 July 2021.

[13] Labbok MH, Wardlaw T, Blanc A (2006). Trends in exclusive breastfeeding: findings from the 1990s. J Hum Lact.

[14] World Health Organization:Breastfeeding. https://www.who.int/health-topics/breastfeeding#tab=tab_1. Accessed 20 Feb 2021.

[15] Martín-Iglesias S, Santamaría-Martín MJ, Alonso-Álvarez A (2018). Effectiveness of an educational group intervention in primary healthcare for continued exclusive breast-feeding: PROLACT study. BMC Pregnancy Childb..

[16] Schilder AG, Chonmaitree T, Cripps AW (2016). Otitis media Nat Rev Dis Primers.

[17] Brennan-Jones CG, Eikelboom RH, Jacques A (2017). Protective benefit of predominant breastfeeding against otitis media may be limited to early childhood: results from a prospective birth cohort study. Clin Otolaryngol.

[18] Bowatte G, Tham R, Allen KJ (2015). Breastfeeding and childhood acute otitis media: a systematic review and meta-analysis. Acta Paediatr.

[19] Chantry CJ, Howard CR, Auinger P (2006). Full breastfeeding duration and associated decrease in respiratory tract infection in US children. Pediatrics.

[20] Usonis V, Jackowska T, Petraitiene S (2016). Incidence of acute otitis media in children below 6 years of age seen in medical practices in five East European countries. BMC Pediatr.

[21] Abrahams SW, Labbok MH (2011). Breastfeeding and otitis media: a review of recent evidence. Curr Allergy Asthma Rep.

[22] Ajetunmobi OM, Whyte B, Chalmers J (2015). Breastfeeding is associated with reduced childhood hospitalization: evidence from a Scottish Birth Cohort (1997–2009). J Pediatr.

[23] Teele DW, Klein JO, Rosner B (1989). Epidemiology of otitis media during the first seven years of life in children in greater Boston: a prospective, cohort study. J Infect Dis.

[24] Duffy LC, Faden H, Wasielewski R (1997). Exclusive breastfeeding protects against bacterial colonization and day care exposure to otitis media. Pediatrics.

[25] Li R, Dee D, Li CM (2014). Breastfeeding and risk of infections at 6 years. Pediatrics.

[26] Chonmaitree T, Trujillo R, Jennings K (2016). Acute otitis media and other complications of viral respiratory infection. Pediatrics.

[27] Karunanayake CP, Albritton W, Rennie DC, et al . Ear infection and its associated risk factors in first nations and rural school-aged Canadian children. Int J Pediatr. 2016. https://pubmed.ncbi.nlm.nih.gov/26977160/. 10.1155/2016/1523897 PMC4764758 26977160

[28] Uhari M, Mäntysaari K, Niemelä M (1996). Meta-analytic review of the risk factors for acute otitis media. Clin Infect Dis.

[29] Daly KA, Giebink GS (2000). Clinical epidemiology of otitis media. Pediatr Infect Dis.

[30] Alsalam AO, Yassen ZM, Buraa MF (2018). Risk factors of acute otitis media among children in Mosul. مجلة دراسات موصلیة.

[31] McNiel ME, Labbok MH, Abrahams SW (2010). What are the risks associated with formula feeding? A re-analysis and review. Birth.

[32] Nielsen S, Nielsen DS, Lauritzen L (2007). Impact of diet on the intestinal microbiota in 10-month-old infants. J Pediatr Gastr Nutr..

[33] Kramer MS, Guo T, Platt RW (2003). Infant growth and health outcomes associated with 3 compared with 6 mo of exclusive breastfeeding. Am J Clin Nutr.

[34] Boone KM, Geraghty SR, Keim SA (2016). Feeding at the breast and expressed milk feeding: Associations with otitis media and diarrhea in infants. J Pediatr.

